# *Dientamoeba fragilis* prevalence coincides with gastrointestinal symptoms in children less than 11 years old in Sweden

**DOI:** 10.1007/s10096-015-2442-6

**Published:** 2015-07-15

**Authors:** J. Ögren, O. Dienus, S. Löfgren, I.-M. Einemo, P. Iveroth, A. Matussek

**Affiliations:** Clinical Microbiology Laboratory, County Hospital Ryhov, 55185 Region Jönköping County, Sweden; Department for Control of Communicable Diseases, Region Jönköping County, Sweden

## Abstract

*Dientamoeba fragilis* is a protozoan with a debated role in gastrointestinal (GI) disease. Although correlated to GI symptoms, no virulence factors have been described. In this study, we evaluated the cause of GI symptoms in children at two schools, with children aged 1 to 10 years, in the county of Jönköping, Sweden. *D. fragilis* infection correlated to GI symptoms in children and *Enterobius vermicularis* correlated to *D. fragilis* infection.

## Introduction

*Dientamoeba fragilis* is a protozoan suspected of causing gastrointestinal (GI) symptoms [1, 2], although its aetiological role in GI disease is controversial [3–6]. The reported prevalence of *D. fragilis* in individuals suffering from GI symptoms varies from 1 to 70 % [4, 7, 8]. No difference in the prevalence of *D. fragilis* has been found in patients suffering from irritable bowel syndrome [6] or children with chronic abdominal pain [9] compared to individuals without symptoms. The life cycle of *D. fragilis* and its mode of transmission are not fully understood and a faecal–oral transmission seems unlikely [10]. It has been hypothesised that *Enterobius vermicularis* may serve as a vector for *D. fragilis* and, recently, *D. fragilis* DNA has been detected in *E. vermicularis* eggs [11].

Traditionally, parasites are detected by microscopy of concentrated unfixed or fixed faecal samples [12]. However, as trophozoites of *D. fragilis* rapidly degenerate outside the host, probably due to the lack of a cyst form [2, 13], a prompt fixation of faecal samples is essential. Also, concentration should be omitted and stained permanent smears or direct wet mounts should be used for the detection of *D. fragilis* trophozoites [12, 14].

Since the introduction of polymerase chain reaction (PCR), the detection of *D. fragilis* has increased [4, 7, 8]. In this study, we use a multiplex PCR assay modified for sodium acetic acid formaldehyde (SAF)-fixated samples to allow comparison with microscopy results on the same samples.

In March 2012, a school nurse alerted the Department for Control of Communicable Diseases in Jönköping County, Sweden, since many children at the school complained of GI symptoms.

We investigated possible infectious causes of GI symptoms in these children. In addition, a multiplex PCR for parasites on SAF-fixated faecal samples was established.

## Materials and methods

### Study populations and GI symptoms

Faecal samples (*n* = 299) were obtained from school children (*n* = 146; 1 to 10 years old) and members of the staff (*n* = 16) at the school where children complained of GI symptoms (school A). Sticky tape tests for *E. vermicularis* detection were obtained from 47 children. Faecal samples from parents (*n* = 123) and siblings (*n* = 14) were also included. For comparison, faecal samples (*n* = 89) and sticky tape tests (*n* = 33) were obtained from children aged 1 to 10 years from another school (school B) in the county. In addition, the results from clinical samples (*n* = 7684) submitted for parasitological investigation to the Clinical Microbiology Laboratory, Jönköping, Sweden in 2012 were included.

Parents to children answered a questionnaire for the epidemiological investigation of GI diseases. Information on GI symptoms [abdominal pain (recurring and/or lasting more than 2 weeks), diarrhoea, nausea or constipation] within 3 months prior to testing was registered. Information regarding GI symptoms was not available for the clinical samples.

### Microscopy for parasites

Faecal samples were immediately fixed in SAF. Samples were then homogenised and sifted, and half of the faecal suspension was concentrated by ethyl acetate treatment. Microscopy (100× and 400×) was done on concentrated material, on direct wet mounts from unconcentrated material and on sticky tape tests.

### Multiplex real-time PCR for parasites

An SAF-fixated faecal solution (1000 μL) was centrifuged at 10,000 × g for 1 min. Pellets were then washed with phosphate-buffered saline (PBS) pH 7.4 for 1 min, followed by another centrifugation step. Pellets were suspended in a mixture of 280 μl AL lysis buffer and 20 μL proteinase K (Qiagen, Hilden, Germany), and incubated at 56 °C for 1 h with gentle agitation. Suspensions were then frozen at −196 °C for 30 min and, finally, heated at 98 °C for 15 min. DNA extraction was done with a MagAttract DNA Mini M48 kit (Qiagen) in an M48 instrument (Qiagen). Real-time PCR for parasite detection was done with the LightCycler 480 II instrument (Roche Diagnostics GmbH, Mannheim, Germany), according to Verweij et al. [7, 15], modified by the inclusion of *Entamoeba dispar* [16].

### Analysis of faecal samples

Samples from 30 children aged 6 to 8 years in school A were also analysed for the presence of GI pathogens, including standard faecal culture, PCR for norovirus, sapovirus [17] and enterohaemorrhagic *Escherichia coli* (EHEC) [18], and antigen detection for rotavirus and adenovirus (Coris BioConcept Combi-Strip, Gembloux, Belgium). All samples from schools A and B were analysed for parasites by multiplex PCR and microscopy, including direct wet mounts for trophozoite detection. Clinical samples were exclusively analysed by microscopy.

## Results

### GI bacterial and viral pathogens

In none of the 30 samples were *Campylobacter* spp., *Salmonella* spp., *Shigella* spp., *Yersinia* spp., EHEC, rotavirus, adenovirus or norovirus detected.

### Parasites detected by microscopy

*D. fragilis* trophozoites were detected in direct wet mounts of unconcentrated faecal samples in 60, 60 and 15 % of samples from school A, school B and in clinical samples, respectively (Table 1). *E. vermicularis* eggs were detected in 13 out of 47 (28 %) sticky tape samples from school A and in 8 out of 33 (24 %) from school B. The prevalence of *D. fragilis* was 95 % (20 out of 21) in those positive for *E. vermicularis* and 69 % (41 out of 59) in those negative for *E. vermicularis* (*p* = 0.03). In concentrated faecal samples, *Blastocystis hominis* was found in nine children and 30 adults at school A and in four children from school B; no other pathogenic parasites or helminths were found. In the clinical samples, 30 *E. histolytica*/*dispar*, 90 *Giardia intestinalis*, 29 *Cryptosporidium* spp., 55 helminth eggs or larvae, and 787 *B. hominis* were found.

**Table 1:** Age-stratified prevalence (%) (number of positive/total number of samples) of *Dientamoeba fragilis* detected by microscopy

	Total	0–5 years	6–10 years	11–15 years	≥16 years
School A	60 (180/299)	45 (32/70)	79 (60/76)	50 (7/14)	58 (81/139)
School B	60 (53/89)	43 (13/30)	68 (40/59)	n.a.	n.a.
Clinical samples 2012	15 (1209/7684)	18 (210/1176)	48 (410/847)	20 (125/613)	9 (464/5048)

*n.a.* not analysed

### Prevalence of *D. fragilis* and recovery by PCR

The age-stratified prevalence of *D. fragilis* detected with microscopy is shown in Table 1. There was no difference in *D. fragilis* prevalence in samples from school A and school B. The prevalence in samples from the age group 6–10 years was higher at schools A and B and in clinical samples, compared to all the other age groups studied (*p* < 0.000 to *p* = 0.02). The prevalence of *D. fragilis* in individuals over 15 years old in school A (parents, staff and siblings) was higher compared to clinical samples (*p* < 0.0000).

All samples positive for *D. fragilis* by microscopy were also positive by PCR. Out of the total of 388 samples, *D. fragilis* was detected in 233 samples by microscopy and in 281 samples by PCR, respectively. This correlates to an increased recovery of 20 % (*p* < 0.000).

### Relation between GI symptoms and *D. fragilis* infection

In school A, 102 questionnaires out of 155 were answered and in 76 children (75 %), one or more of the symptoms were documented. In school B, 60 out of 89 questionnaires were answered and in 32 children (53 %), one or more symptoms were documented. The prevalence of *D. fragilis* was higher in those having GI symptoms compared to children without symptoms (Table 2).

**Table 2:** Relation between gastrointestinal (GI) symptoms and *Dientamoeba fragilis* infection

	*n*	GI symptoms		No GI symptoms		*p*-Value*
		*D. fragilis*		*D. fragilis*		
		Yes	No	Yes	No	
School A	102	66	10	4	22	<0.000
School B	60	26	6	6	22	<0.000

*Comparing the prevalence of *D. fragilis* DNA in those with and without symptoms

## Discussion

In this study, we found that *D. fragilis* infection in children under the age of 11 years coincided with GI symptoms. This was not found in individuals over 10 years of age (data not shown). These findings are in agreement with previous studies on children [5] and adults [6], and suggest a role of *D. fragilis* in GI disease in children. The prevalence of *D. fragilis* was higher in the two schools studied compared to clinical samples. Interestingly, the prevalence in adults at school A was equally high as that in the children, whereas in clinical samples, the prevalence was less than 10 %. This is in agreement with previous findings of a high prevalence of *D. fragilis* in adults with close contact to children [4].

A drawback of this study was that GI symptoms were evaluated by reviewing questionnaires answered by parents and with no gradient scale to describe the severity of the symptoms. However, at school A, the symptoms were severe enough to alert the school nurse to contact the Department for Control of Communicable Diseases.

We found a higher prevalence of *D. fragilis* in children simultaneously infected with *E. vermicularis* compared to children with no *E. vermicularis* eggs detected. The results confirm previous studies based on the analysis of sticky tape test for the detection of *E. vermicularis* [19] but contradicts results from studies based on the analysis of faecal samples for *E. vermicularis* detection [20]. Our results indicate a role for *E. vermicularis* in the transmission of *D. fragilis*. Recently, *D. fragilis* DNA was detected in *E. vermicularis* eggs [11, 21] and in a study on metronidazole treatment of *D. fragilis* infection, *E. vermicularis* co-infection increased the risk of post-treatment *D. fragilis* infection [22]. Our finding of an equally high prevalence of *D. fragilis* in adults in contact with infected children raises the question regarding the routes of transmission to adults. Recently, a cyst form of *D. fragilis* has been detected in faeces from humans, but a possible faecal–oral transmission has not been documented [23, 24].

In this study, we found that a multiplex PCR, adapted to SAF-fixated faecal samples, increased the detection of *D. fragilis* by 20 % compared to microscopy.

In conclusion, our findings add to the opinion that *D. fragilis* might have an aetiological role in GI symptoms in children, and that *E. vermicularis* has a possible role in the transmission of *D. fragilis*.
